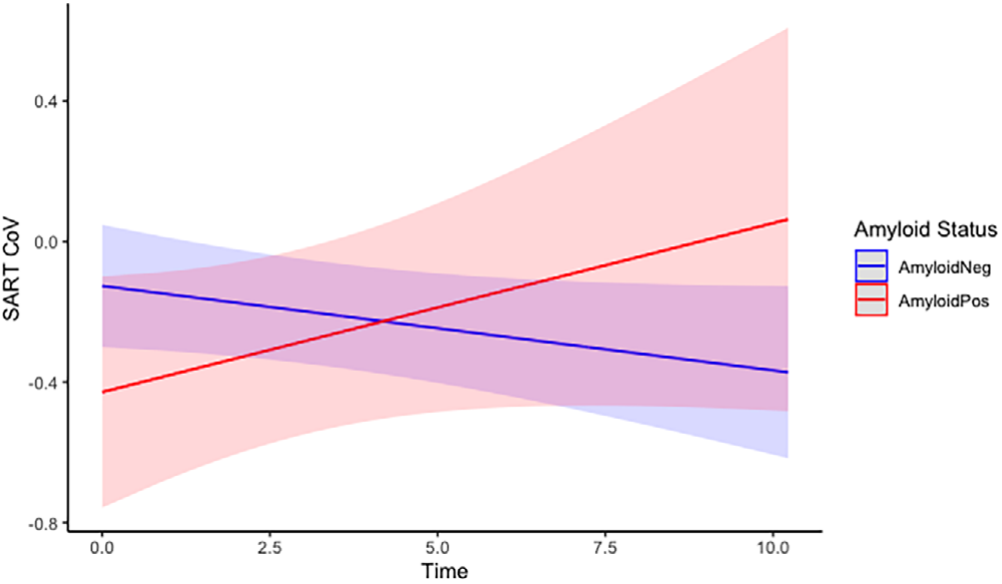# Variability in Reaction Time Predicts Transition to Mild Alzheimer Disease and is Sensitive to CSF Biomarkers in Cognitively Normal Older Adults

**DOI:** 10.1002/alz.087068

**Published:** 2025-01-03

**Authors:** Matthew S. Welhaf, David A. Balota, Suzanne E. Schindler, John C. Morris, Jason J. Hassenstab, Andrew J. Aschenbrenner

**Affiliations:** ^1^ Washington University in St. Louis, St. Louis, MO USA; ^2^ Department of Neurology, Washington University School of Medicine, St. Louis, MO USA; ^3^ Washington University in St. Louis, School of Medicine, St. Louis, MO USA; ^4^ The Charles F. and Joanne Knight Alzheimer Disease Research Center, St. Louis, MO USA; ^5^ Knight Alzheimer Disease Research Center, St. Louis, MO USA; ^6^ Washington University St. Louis, St. Louis, MO USA; ^7^ Washington University School of Medicine in St. Louis, St. Louis, MO USA

## Abstract

**Background:**

The ability to detect cognitive impairment from Alzheimer Disease (AD) in its earliest possible symptomatic stage is a highly desirable characteristic for neuropsychological measures. Because early cognitive changes are often subtle, measures with high sensitivity are of great importance. Variability in attention, often assessed using reaction time (RT) tasks, have been shown to discriminate between cognitively normal older individuals with and without positive AD biomarkers and is correlated with biological markers of neurodegeneration. However, what is less clear is how well measures of RT variability predict later development of cognitive symptoms of AD and whether common AD biomarkers predict declines in attention.

**Method:**

Cognitively normal older adults (N = 478, baseline age = 66.8 ± 8.9 years) completed a go/no‐go task, the Sustained Attention to Response Task (SART). Participants were instructed to respond to all numbers except for the digit “3”. Variability in RT was measured using the Coefficient of Variation (CoV) to correct “go” trials. Cognitive status was assessed using the Clinical Dementia Rating® (CDR®). Logistic regression was used to test for the sensitivity of RT CoV in predicting eventual conversion to CDR > 0, adjusted for baseline age, education, and gender. A subset (N = 197) of participants had CSF Aβ42/Aβ40 within 3 years of the first SART assessment and 2+ visits allowing for testing longitudinal changes. Mixed effect models were used to track longitudinal change in RT CoV based on amyloid status.

**Result:**

Greater RT CoV (and thus poorer attention) predicted later conversion to CDR > 0 (p = 0.008), after accounting for baseline levels of education, age, and gender. There was a significant interaction between amyloid status and time (p = 0.048). Increases in RT CoV over time occurred for individuals designated as being amyloid positive (Aβ42/Aβ40<0.0673), but not for those initially identified as amyloid negative (see Figure 1).

**Conclusion:**

Variability in RT is a significant predictor of later development of cognitive symptoms associated with AD. Longitudinal changes in RT CoV are also sensitive to baseline amyloid status. These findings highlight the sensitivity of within‐person fluctuations of attention as a cognitive biomarker for AD.